# Infection prevention and control during the COVID-19 pandemic: challenges and opportunities for Kenyan public hospitals

**DOI:** 10.12688/wellcomeopenres.16222.1

**Published:** 2020-09-10

**Authors:** Michuki Maina, Olga Tosas-Auguet, Mike English, Constance Schultsz, Jacob McKnight

**Affiliations:** 1 Health Services Research Group, KEMRI Wellcome Trust Research Programme, Nairobi, Kenya; 2 Department of Global Health, Amsterdam UMC, University of Amsterdam, Amsterdam, The Netherlands; 3 Nuffield Department of Medicine, University of Oxford, Oxford, UK; 4 Amsterdam Institute for Global Health and Development, University of Amsterdam, Amsterdam, The Netherlands

**Keywords:** Water Sanitation and Hygiene, Infection Prevention and Control, COVID- 19

## Abstract

**Background: **Infection prevention and control, and water sanitation and hygiene have an essential role in ensuring the quality of care and patient outcomes in hospitals. Using a modification of the World Health Organization’s water sanitation and hygiene facility improvement tool, we undertook assessments in 14 public hospitals in Kenya in 2018. The hospitals received written feedback on areas where they could make improvements. Following the first confirmed cases of COVID-19 in Kenya, we were drawn to ask whether the results of our pre-pandemic survey had led to action, and whether or not the threat of COVID-19 had focused more attention on infection prevention and control and water sanitation and hygiene.

**Methods: **Using a semi-structured interview guide, we carried out phone interviews with key hospital leaders in 11 of the 14 hospitals. The data were transcribed and coded into thematic areas. We draw on these interviews to describe the status and awareness of infection prevention and control.

**Results: **The infection prevention and control committee members are training health workers on infection prevention and control procedures and proper use of personal protective equipment and in addition, providing technical support to hospital managers. While some hospitals have also accessed additional funds to improve infection prevention and control, they tended to be small amounts of money.  Long-standing challenges with supplies of infection prevention and control materials and low staff morale persist.  Crucially, the reduced supply of personal protective equipment has led to fear and anxiety among health care personnel.

**Conclusions: **As funds are mobilised to support care for COVID-19, we ask that funds prioritise infection prevention and control measures. This would have a profoundly positive effect on within hospital virus transmission, patient and staff safety but also lasting benefits beyond the COVID-19 pandemic.

## Introduction

Improving water sanitation and hygiene (WASH) and infection prevention and control (IPC) in medical facilities is crucial to the fight against COVID-19 in all countries, but particularly so in those countries with long-standing problems in maintaining effective infection control ^1^. Hospitals are now thought to have been significant sites for COVID-19 infection spread in some of the countries worst affected by the pandemic ^2^. Ensuring effective infection control in hospitals protects patients and health workers, improving worker confidence and morale. Maintaining a healthy workforce is essential to the COVID-19 response and the provision of all other health interventions ^3^. 

We conducted a WASH survey of 14 Kenyan hospitals in 2018. We adapted the World Health Organisation's WASH facility improvement tool to explore performance differences between hospitals and between wards in individual hospitals ^4^. We found a wide degree of performance variation and particular problems in hand hygiene and waste management infrastructure. We also proposed accountability structures to facilitate improvements in WASH ^5, 6^. All the 14 original hospitals had an IPC focal person who was trained to conduct WASH-FAST surveys and had surveyed at least four hospitals, including their own; each hospital received detailed site-specific feedback on their results. We hoped that this feedback and the training given to local focal points might prompt local post-survey WASH and IPC improvement efforts.

The first confirmed case of COVID-19 in Kenya was announced on 12 ^th^ March 2020. This was accompanied by extensive media coverage and citizen education on COVID-19, including the importance of social distancing and proper hand hygiene. At this point, the main policy measures implemented by the government included quarantine of travellers returning to Kenya, the suspension of public gatherings and the setting up of a national COVID-19 isolation unit in Nairobi ^7^. No restriction of movement or curfews were executed at this point. Following the first confirmed cases, we were drawn to ask what actions had occurred, and whether or not the threat of COVID-19 was focusing more attention on IPC and WASH. On 2 ^nd^ April 2020, we contacted the hospital staff who previously led the WASH surveys. By this date, there were 110 confirmed cases around the country, and the main government interventions included restriction of movement and a nationwide dusk to dawn curfew. The government, through the regional governments, had embarked on setting up COVID-19 isolation units in multiple county hospitals ^8^. As April proceeded, Kenya requested World Bank funding for these isolation units, for training and for the purchase of ventilators to improve intensive care capacity ^9^.

## Methods

### Interview data collection

We conducted semi-structured phone interviews in April and May 2020. Each interview lasted approximately 20–30 minutes, with the consent of 11 representatives of the original 14 hospitals, (the semi-structured interview template can be found in the online data repository). The respondents were medical officers, dentists, pharmacists, non-physician clinicians, nurses, and public health officers. Of these, one was a hospital director, and another was in charge of hospitals across the administrative region (sub-county). The interviewees comprised a balanced mix of gender, age and experience. The semi-structured interview instrument used was informed by data collected from the previous surveys. The interview guide was prepared by all the authors, who consisted of a mix of clinicians, epidemiologists, health system experts and medical anthropologists. The authors are also very familiar with the Kenyan health system and IPC context and were all actively involved in the previous IPC/WASH surveys in 2018 ^5, 6^. The first author conducted the phone interviews. The first author, who is a Kenya doctor, also led the original surveys and is familiar with the health system context. . Field notes were not taken. Background information concerning the study and interview was availed to these interviewees before consenting. As interviews were conducted over telephone, all respondents gave verbal informed consent for the interviews.

### Analysis of data obtained from interviews

We sought to understand if any changes or improvements had occurred within the hospital for each area we had previously analysed ^5, 6^. We then asked how the present COVID-19 pandemic was affecting efforts to improve the WASH infrastructure and IPC, how the facility was preparing for COVID- 19 patients and how the pandemic had affected hospital and health worker activities. The audio files of each interview were transcribed and uploaded into NVivo 12 and kept on an encrypted laptop. The first author coded the transcripts independently before discussing the codes and agreeing on combined axial codes and thematic areas with the last author. The findings from these themes illustrated using quotes. No repeat interviews were conducted. A Consolidated criteria for Reporting Qualitative research (COREQ) checklist was completed and is included in the data repository ^10^. Summary data are available, see *Underlying data* ^10^.

### Ethical approval

This study received approval from the Oxford Tropical research ethics committee (OXTREC) from the University of Oxford (Ref: 525–17) and the Kenyan Medical Research Institute (Ref: KEMRI/SERU/CGMR-C//086/3450).

## Results

### Infection control status and awareness improved

Our original survey had noted the low performance of WASH and infection control. Previously, staff responsible for hygiene issues contrasted infection control committees with those of more prestigious domains, such as the medicines committee, and complained that members did not attend meetings. Since the emergence of COVD-19, this has changed and the roles played by the IPC committees have gained more focus and importance.

*"Actually, right now people are more responsive, previously you call people on things to do with IPC and all that people used to take it for public health workers and all that. But now we are even having the consultants attending."* IPC Committee Member.

IPC committees are also now being asked to offer technical advice to managers.

*"But now we also do have supervision. The committee does the supervision within the hospital, and then reports whatever we have agreed and seen to the CEO."* Hospital IPC coordinator. 

Hence, infection control has become an urgent priority, and the interviews revealed hospital staff at all levels are now concerned about IPC best practice. This may be due to the fear of contracting COVID-19 at work, and it was clear that these concerns are leading to a great deal of anxiety:

*"There is a lot of fear initially the time we were starting out. And after the once who have gone through things change for example initially, they could say they can't see patients without an N95 mask, but after the training on which masks to be worn where people become more confident. The element of fear and anxiety is still there, but the ones who attend the training it becomes less."* IPC Leader.

### Positive changes

The interest in preparing for COVID-19 has resulted in positive actions in several areas: In some of the facilities, this has offered an opportunity to increase the IPC supplies and infrastructure including hand hygiene facilities in the hospital, albeit some of these are temporary. 

*"I believe that after this we will go far, we never had sanitisers before, these days we have sanitiser."* Hospital IPC leader.

Where previously budgets for IPC activities were non-existent, in some hospitals some funds are now available specifically for IPC activities.

*"I don't know where the med sup [Medical Director] got some little money. Almost USD 1500, he was telling us to come up with a budget. We sat last week. And we have given the proposal to him."* IPC Committee Leader.

Some of the facilities reported an improvement in the cleanliness standards. This was mainly through reallocation of cleaning staff and increased supervision in critical areas of the hospitals to ensure these were regularly cleaned, and waste management was improved.

*"And then the cleanliness, we improved on the supervision. Yeah, we got another public health officer. And we got at least two supervisors for the cleaning company. And then with the supervision, there is some, there is an improvement."* IPC Lead.

Some of the facilities reported significant improvement in WASH infrastructure including fixing of hand hygiene stations;

*"The sinks even if they are not fully functional, maybe we are over 90% there. Because at least you can repair one and it gets spoilt. But I'm telling you we are very far. That one we have done it."* IPC Lead.

### Ongoing IPC challenges

While the respondents noted areas of improvement, the overall assessment of our respondents suggested that long-standing issues had not been adequately resolved. These include challenges with waste management, infrastructure and funding. Some of the areas where challenges persisted included in infrastructure where, for example, poor quality fixtures of hand hygiene (taps and pipes) led to frequent breakages and leaks. Other areas with long-standing challenges were waste management and funding for IPC activities. All these play a critical role in service delivery and staff morale.

*"The other challenge is that currently, our incinerator is down. So, we are not able to offer incineration services. Both in house and even anyone from outside. So, what we are doing right now we are disposing our waste off sight."* IPC Committee Leader.

*"But of course, here, staff morale has gone down because of the issue of salaries – it's so delayed. Like now, we have not even got our salaries you can imagine."* Hospital Manager.

Newer challenges have also emerged. Though some of the IPC supplies are present, in some facilities, the supply diminishes rapidly due to theft and misuse.

*"Even the soap, you put it outside there, and the following day even the container is not there. Yes, sometimes we put the soap there and the relatives of the patients when they come in, they think that maybe that is the soap they should be using for washing clothes. They drain it all out in the basins when they come across it. They are taking it away."* IPC lead.

### Health worker safety

In addition to these long-standing issues, a host of new issues related to the pandemic response have been raised, and most prominent among these are concerns about the safety of health workers. These include challenges in the acquisition of personal protective equipment (PPE) for both clinical and support staff.

*"Right now, it's not adequate because we are yet to get the gowns, the masks and the gloves we have. But the gowns which I think we really need we don't have. But at the designated areas they have some."* IPC Leader.

The lack of PPE is strongly related to levels of anxiety and concerns about the ability of the workforce to do its job:

*"Yes, we are very, very anxious. We are so worried about ourselves, our safety before even the patients because we have families definitely. So, there is this sort of some attitude."* Doctor.

*"Everybody is worried. Everybody, but if they provide people with enough PPE'S, nobody is there to worry. People are ready to work."* IPC Leader.

### Health seeking behaviour in the COVID-19 pandemic

In terms of patients seeking care for other ailments, we noted the numbers have significantly reduced in the facilities. However, the hospitals that serve large populations still have to contend with crowding. This is a challenge in reducing hospital-acquired infections.

*"The management had decided to halt outpatient clinics; unfortunately, we still have a problem with that. like today is our Gynaecology Outpatient Clinic, so we expected we wouldn't have any patients, but apparently, we were so disappointed when we found like about thirty patients and you know they are all crammed together, there is no even social distancing or anything"* Medical Doctor

We contrast this to other facilities where numbers are worryingly low. The concern is some of these patients will lack much-needed care. This may, in the long run, affect the gains made in reducing the infant and maternal mortalities in the region.

## Key insights

### Huge opportunity for improvements due to status change and interest.

The considerable importance of WASH and IPC in addressing COVID-19 provides a real opportunity for improvement of IPC in health facilities. The existing structure of IPC committees and coordinators, and public health officers, could offer a real opportunity for highly efficient use of COVID-19 funds. A bottom-up approach would ensure funds are allocated where they are needed. Some hospitals have embraced this and are making positive changes, even with limited resources.

*"…I was given the responsibility of like a coordinator; I usually go around on a daily basis. You decide today to go to the medical department, maybe the following day you can go to maternity, you can go to paediatrics. You look at the way they are segregating you find that they are not doing it properly you just correct them."* IPC Lead.

### Structural weaknesses, long-standing issues need to be prioritised and dealt with urgently.

The issues of lack of crucial infrastructure still exist. These include poor waste disposal, plumbing to guarantee water supply in the wards and proper ventilation. Improving plumbing and access to appropriate sinks are some of the interventions that can be effected quickly. Others may include long term investments like improving ventilation within the hospitals by the installation of exhaust fans. Other long-term investments would consist of setting up/building fully-fledged isolation units.

However, these investments need to be prioritised in budget allocations as they are essential for the appropriate management of COVID-19 and other infectious conditions which are prevalent in our region. Investing in IPC and WASH would greatly complement the efforts being put to improve the availability of oxygen and critical care support in some of the hospitals in our survey and across the country.

### Health systems challenges

The greatest risk COVID-19 poses in our context is a health system collapse. In most facilities, though the patient load has significantly reduced, there is still a need to ensure the health system does not get overwhelmed by COVID-19. Health system collapse and closure of services such as maternal and child health would result in tragic increases in maternal and child mortality ^11^.

There have been numerous reports of frontline health workers globally contracting COVID-19 in the line of duty and some due to lack of proper PPE ^12, 13^. If health workers do not feel safe while offering services, some may choose to stay away or take industrial action, including strikes. Strikes have been frequent in Kenya and the region with devastating effects, and there are ongoing negotiations to avert a looming health worker strike in the country ^14, 15^. Motivating staff and ensuring safe working conditions, including PPE, training and treatment if they fall ill needs to be a priority at this time. With already low staff numbers, staff staying off work due to illness or fear of COVID-19 as a result of weak IPC structures would easily overwhelm service delivery within hospitals.

We can seize this moment to make things better not only during this pandemic but for years to come.

*"but again, COVID- 19 is a blessing in disguise it's not the right phrase to use but I think this COVID coming, it's a wakeup call to all these people who were like taking things for granted. Our health system is in trouble; it has been in trouble all through, now at least they are seeing."* Medical Doctor.

## Conclusion

Effective treatment of COVID-19 relies on effective IPC and WASH and funds must be immediately directed towards this in low- and middle-income countries such as Kenya where performance is often poor. These gains could have long-lasting positive effects for other huge issues facing hospitals such as antimicrobial resistance and hospital-acquired infections. We believe that investing in IPC should be prioritised in advance of setting up critical care units as part of the COVID-19 response ^9^. If we wish to mitigate the effects of COVID-19 on health systems and avert the collapse of an already stretched health system, IPC and WASH should be recognised as a major policy priority. Staff safety should be prioritised by availing proper PPE for health workers in their line of duty and during procedures that are aerosol-generating to ensure they remain disease-free. 

## Data availability

### Underlying data

Harvard Dataverse: Replication Data for: Water Sanitation and Hygiene in Kenyan Hospitals. https://doi.org/10.7910/DVN/IJUWWR ^10^.

This project contains the following underlying data:

- WASHKenya.tab. (Summary data for this study.)
- codebook_Michuki_WASH.pdf. (Codebook for the above data file.)

Access to the transcripts is restricted since they contain identifying information about participants. Applications for access of the study transcripts can be made through the Data Governance Committee ( dgc@kemri-wellcome.org). Further details are available at https://kemri-wellcome.org/about-us/. This contains guidelines for how to apply for access and an application form.

### Extended data

Harvard Dataverse: Replication Data for: Water Sanitation and Hygiene in Kenyan Hospitals. https://doi.org/10.7910/DVN/IJUWWR ^10^.

File ‘MainaetalPC_supplement.docx’ containsthe questions asked to each particiapnt.

### Reporting guidelines

Harvard Dataverse: COREQ checklist for ‘Infection prevention and control during the COVID-19 pandemic: challenges and opportunities for Kenyan public hospitals’. https://doi.org/10.7910/DVN/IJUWWR ^10^.

Data are available under the terms of the Creative Commons Attribution 4.0 International license (CC-BY 4.0).
