# Supplementary material for: Infection prevention and control during the COVID-19 pandemic: challenges and opportunities for Kenyan public hospitals
Source: Wellcome Open Res. 2020 Sep 10;5:211. [Version 1] doi: 10.12688/wellcomeopenres.16222.1 (PMC7645384; doi:10.12688/wellcomeopenres.16222.1)
Supplement: Peer review history

# Peer review: Reviewer response for version 1

**DOI:** 10.21956/wellcomeopenres.17815.r40688

**Published:** 2020-11-05

**Authors:** Lydia Okutoyi

**Affiliations:** 1 Department of Quality and Patient Safety, Kenyatta National Hospital, Nairobi, Kenya

1. General comments: The study conducted among health facilities that had previously been part of a WASH assessment by the first author. This gave a basis for having a prior assessment of the WASH related structures and processes before the pandemic. Given the current situation phone interviews were an easier option and safer option too. I would have liked to know whether the same staff were interviewed, 2 years is a long time. Among the staff who participated in the previous WASH study, getting a realist assessment of what changes happened after the WASH, and which changes are attributable to COVID 19 response. I would be interested to know the match between the WASH tool used for assessment and the guiding questions for this study.
  Qualitative methods enabled better understanding of the various experiences with regards to staff safety, leadership and un-intended effects of having scarce recourses available in the facilities. The coding for themes was well done.
2. Provide answers to our mandatory reviewer questions: The work is accurate as provided and cites existing literature. The findings are generally replicable in Kenyan public sector. Having lead the IPC efforts in KNH I concur with the findings. They are generally replicable as much as qualitative survey may depict peculiar experiences. The documented experiences have been reported in media and some at a public hospital I work at.. The study design is appropriate in the COVID Era.
3. Generally Approved. Consider the comments in the text above.

Is the work clearly and accurately presented and does it cite the current literature?

Yes

If applicable, is the statistical analysis and its interpretation appropriate?

Yes

Are all the source data underlying the results available to ensure full reproducibility?

Yes

Is the study design appropriate and is the work technically sound?

Yes

Are the conclusions drawn adequately supported by the results?

Yes

Are sufficient details of methods and analysis provided to allow replication by others?

Partly

Reviewer Expertise:

NA

I confirm that I have read this submission and believe that I have an appropriate level of expertise to confirm that it is of an acceptable scientific standard.

# Peer review: Reviewer response for version 1

**DOI:** 10.21956/wellcomeopenres.17815.r40415

**Published:** 2020-09-22

**Authors:** Angela Dramowski

**Affiliations:** 1 Department of Paediatrics and Child Health, Faculty of Medicine and Health Sciences, Tygerberg Hospital, Stellenbosch University, Cape Town, South Africa

This timely study uses telephonic, semi-structured interviews with hospital representatives to document changes in IPC and WASH programme implementation and performance at 11 Kenyan hospitals. The findings of the survey are contrasted with data reported from a 2018 survey of 14 Kenyan hospitals, which documented widespread shortcomings in WASH and IPC infrastructure and organisation. Using thematic analysis, the authors describe improvements in IPC awareness and programme status, increased provision for hand hygiene and increased facility cleanliness following the emergence of COVID-19 in Kenya. Despite small increases in budgetary allocation to IPC activities, many challenges remained or emerged including waste incineration, theft/removal of IPC consumables and shortages of PPE required for COVID-19. Participants raised concerns regarding health care worker safety during the pandemic and the likelihood of service disruption owing to staff absences (illness and absenteeism due to fear) or even strike action. The authors accurately convey the ongoing challenges to IPC in African healthcare facilities and describe how these have been further amplified by COVID-19. The authors advocate for prioritisation of funds to strengthen IPC and WASH programmes, so that some of the IPC "gains" during the COVID-19 pandemic may have a lasting impact on healthcare quality, patient and staff safety. This is an important manuscript that warrants indexing with no substantive changes required.

Is the work clearly and accurately presented and does it cite the current literature?

Yes

If applicable, is the statistical analysis and its interpretation appropriate?

Not applicable

Are all the source data underlying the results available to ensure full reproducibility?

Yes

Is the study design appropriate and is the work technically sound?

Yes

Are the conclusions drawn adequately supported by the results?

Yes

Are sufficient details of methods and analysis provided to allow replication by others?

Yes

Reviewer Expertise:

Infection Prevention and Control.

I confirm that I have read this submission and believe that I have an appropriate level of expertise to confirm that it is of an acceptable scientific standard.
